# Hypersensitivity to Folic Acid and/or Folinic Acid—A Review of Clinical Cases, Potential Mechanism, Possible Cross-Allergies and Current Diagnostic Options

**DOI:** 10.3390/cimb47080654

**Published:** 2025-08-14

**Authors:** Kinga Lis

**Affiliations:** Department of Allergology, Clinical Immunology and Internal Medicine, Ludwik Rydygier Collegium Medicum in Bydgoszcz, Nicolaus Copernicus University in Torun, ul. Ujejskiego 75, 85-168 Bydgoszcz, Poland; kinga.lis@cm.umk.pl

**Keywords:** folic acid, folinic acid, methotrexate, hypersensitivity, cross-allergy, diagnostics

## Abstract

Folic acid and its derivatives (e.g., folinic acid) are a group of water-soluble compounds collectively known as vitamin B9. Synthetic folic acid is a component of dietary supplements, medications and other pharmaceuticals and fortified foods. Folinic acid (5-formyltetrahydrofolic acid) is the active metabolite of folic acid. It is used to treat vitamin B9 deficiency and as an adjunct to various combination therapies. Hypersensitivity reactions to folic acid or folinic acid are rare and occur following exposure to synthetic folic acid or its derivatives but not on natural folates. In people allergic to folates, cross-reactions are possible following exposure to folic acid analogues (including antifolates, e.g., methotrexate). The mechanism of hypersensitivity to folic acid and/or folinic acid has not been clearly established. Both IgE-dependent and non-IgE-dependent hypersensitivity reactions are likely. It is possible that folic or folinic acid is either an immunogen or a hapten. Diagnosing hypersensitivity to folic/folinic acid is difficult. There are no validated in vitro or in vivo diagnostic tests. The basophil activation test (BAT) appears to be a promising tool for diagnosing folate allergy. The aims of the manuscript were to review published clinical cases of hypersensitivity reactions to folic or folinic acid, potential mechanisms of these reactions and possible cross-allergies, and current diagnostic possibilities of folate hypersensitivity.

## 1. Introduction

Folates (including folic acid and folinic acid) are classified as water-soluble B vitamins. The term “folates” is a general term used to describe a number of vitamins from the B9 family (sometimes also called vitamin B11). The B9 vitamin group includes both synthetic folic acid and its naturally occurring derivatives. Folic acid is a form of vitamin B9 that, due to its chemical stability, is used in vitamin supplement tablets and cell culture media [1]. Folic acid plays a key role in many metabolic processes, including nucleic acid synthesis [2]. As a substance not produced by animals, it must be supplied to the body through food (folates) or supplemented with pharmaceuticals, dietary supplements or vitamin supplements (folic acid) [3,4,5]. Folic acid is also used as a carrier for various drugs and chemotherapeutics, enabling the delivery of folate-bound substances into cells via endocytosis via the folate receptor (FR) [6,7,8,9].

Vitamin B9, especially of natural origin, does not appear to pose a significant risk of inducing a hypersensitivity reaction and is not generally considered a primary cause of allergy. However, it may be a substance worth considering in the diagnostic process for hypersensitivity reactions with a difficult-to-identify causative factor [10].

The aims of the manuscript were to review published clinical cases of hypersensitivity reactions to folic or folinic acid, to present potential etiological mechanisms of these reactions and possible cross-allergies, clinical implications of these reactions and to analyze current diagnostic possibilities of folate hypersensitivity.

This narrative review included publications on hypersensitivity/allergy to folinic acid and/or folic acid published up to July 2025 (in English and non-English). Publications were searched for keywords in PubMed, Google Scholar, etc., and in popular search engines (e.g., Google) using the terms “hypersensitivity” or “allergy” or “anaphylaxis” and “folic acid” or “folinic acid” and by analyzing the references of the reviewed publications. No artificial intelligence (AI) was used for data retrieval and analysis.

## 2. Folic Acid and Folinic Acid

### 2.1. History of Discovery and General Information

The discovery of folate is owed to Lucy Wills’s (1888–1964) long-standing research into the cause of severe anemia in pregnant women. Between 1929 and 1931, Wills investigated the cause of severe anemia in pregnant women in Bombay, India. Clinical symptoms of pernicious anemia in pregnancy included swelling of the face, ankles and feet, weakness, hypotension, intermittent fevers, sore mouth and tongue, and diarrhea. In extreme cases, it led to death. Microscopic examination of blood smears revealed macrocytosis [11,12,13,14]. Although the symptoms and appearance of erythrocytes resembled the recently described pernicious anemia [15,16,17], hematological disorders in pregnancy were considered a distinct disease entity [12]. Due to the climatic and sanitary conditions in India, Wills initially focused on the infectious etiology of this disease. After ruling out this possibility, she turned her attention to the nutritional causes of anemia. The concept of nutritional deficiencies causing anemia was linked both to the analogy to the effects of vitamin B12 deficiency and to the results of analysis of women’s diets, which were deficient in both caloric value and quality of the consumed foods. L. Wills’s research led to the discovery of an essential factor of dietetic origin, the deficiency of which results in the occurrence of anemia during pregnancy. Wills identified this factor as present in yeast and liver, which, when introduced into the diet of affected women, effectively treated the symptoms of anemia [18]. Wills published her observations, research results and descriptions of effective therapy in 1930–1931 in a series of papers [19,20,21,22,23,24,25].

This then-new hematopoietic factor, present in yeast and liver, which treated tropical macrocytic anemia in humans, was initially called the “Wills factor.” Further studies of the chemical and biological properties of this substance led to its recognition as a vitamin, initially called vitamin M (from the English word “monkey”) or vitamin B(c) (from the English word “chick”). These names stemmed from the observation that this factor was effective in preventing nutritional pancytopenia in monkeys and in treating experimental anemia in chickens. Folic acid was isolated in 1941 by Herschel Kenworthy Mitchell from spinach leaves [26]. Mitchell then proposed the name folic acid, from the Latin word “folium,” meaning the leaf from which the vitamin was extracted [26,27,28]. Since then, folic acid has been produced by chemical synthesis, microbiological synthesis or metabolic engineering on an industrial scale [29,30,31].

### 2.2. Physical and Chemical Properties, Biological Function, Metabolism

**Figure 1:**
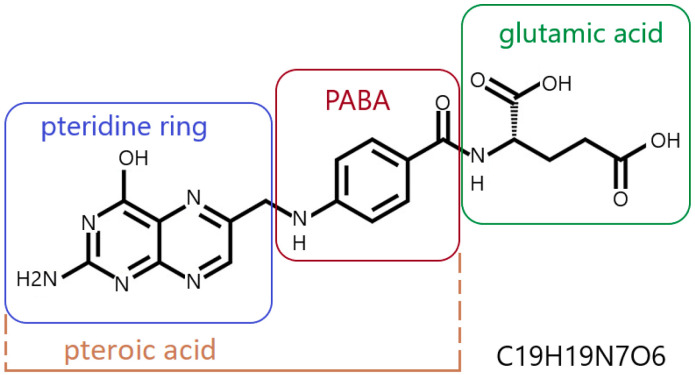
Folic acid—structural formula and empirical formula (Hill notation); PABA—para-aminobenzoic acid; author’s own figure based on [3,7,34,35,36,37,38].

Folic acid (pteroylglutamic acid) is an N-acylamino acid with a molecular weight of 441.4 g/mol. It is a complex chemical compound whose molecule contains three components: a pteridine derivative (2-amino-4-hydroxy-6-methylpteridine), p-aminobenzoic acid (PABA) and glutamic acid (Figure 1) [32,33].

A folic acid molecule can contain more than one glutamic acid residue. It typically occurs as a polyglutamate conjugate, with 2 to 9 glutamic acid residues attached to a pteroyl acid residue (Figure 1) [7,34,35]. Pure folic acid is a synthetic compound, presenting as odorless, orange-yellow needles or platelets. It darkens and carbonizes at temperatures around 250 °C [32]. Under natural conditions, folic acid forms folates, which are derivatives of folic acid with varying degrees of pteridine ring oxidation and varying numbers of glutamic acid residues [6]. Folic acid is synthesized and converted into folates by higher plants, yeasts and some bacteria (including gastrointestinal bacteria). Animals do not synthesize folic acid because they do not produce PABA and do not have the ability to form a pteroyl residue link with glutamate [36,39,40].

**Figure 2:**
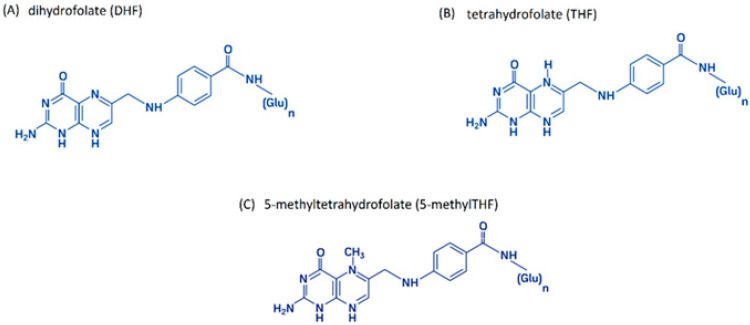
The main biologically active folic acid derivatives: (**A**) dihydrofolate (DHF); (**B**) tetrahydrofolate (THF); (**C**) 5-methyltetrahydrofolate (5-methylTHF); author’s own figure based on [4,41,42,43,44].

Folic acid, regardless of its natural or synthetic origin, is not biologically active. It is only converted into active forms during metabolism. Active forms of folates are formed by the reduction of folic acid to dihydrofolate (DHF) (Figure 2A) and then to tetrahydrofolate (THF) (Figure 2B) in reactions catalyzed by dihydrofolate reductase (DHFR). The main folate found in blood is 5-methyltetrahydrofolate (5-methylTHF) (Figure 2C) [34,41,42,43,44].

Folate circulates in the blood as monoglutamate derivatives. Folate is transported into cells via several pathways: folate receptor (FR)-mediated endocytosis, reduced folate carrier (RFC) or proton-coupled folate transporter (PCFT) [7,9,35,41]. Active folate forms are essential coenzymes that play a key role in numerous metabolic processes ensuring the proper functioning of all body cells (Box 1) [40,45,46,47].

Box 1 **Important processes in which tetrahydrofolic acid participates as a coenzyme involved in the transfer of one-carbon fragments [41,48].**

• Purine and pyrimidine base synthesis (essential for nucleic acid synthesis)
• Nucleotide synthesis
• Protein synthesis
• Phospholipid synthesis
• Protein methylation
• Deoxyribonucleic acid (DNA) methylation
• Remethylation of homocysteine to methionine (methionine cycle)
• Conversion of histidine to glutamic acid

**Figure 3:**
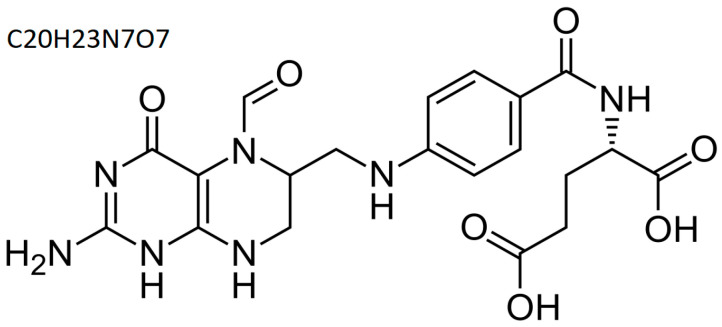
Folinic acid—structural and summary formula; author’s own figure based on [51].

Folinic acid or levofolinic acid (leucovorin) (Figure 3) is an active form of folic acid (5-formyltetrahydrofolic acid) used in the treatment of anemia or as an adjunct to cancer therapy with some chemotherapeutic agents (e.g., methotrexate and 5-fluorouracil) [49,50].

This strategy allows for the delivery of a biologically active form to the body, bypassing endogenous metabolic pathways that may be inactive or intentionally blocked during therapeutic procedures. The use of folinic acid in treatment or as adjunctive therapy accelerates the achievement of the intended therapeutic effects (e.g., in the treatment of anemia) and/or reduces the toxicity of the therapy used (e.g., in cancer treatment) [43,50,51].

Adequate availability of active forms of folate is particularly important during phases of rapid cell division, including periods of rapid growth and development, pregnancy and erythropoiesis [52]. Due to its participation in metabolic processes essential for cell growth and proliferation, as well as metabolic changes occurring in cancer cells aimed at maximizing nutrient utilization (e.g., increased expression of the folate receptor on the cell surface), folic acid supports cancer cell proliferation, which promotes tumor growth and development. It is believed to play a protective role in cancer development [53,54,55]. Some therapeutic strategies used in the treatment of cancer utilize the overexpression of a receptor essential for folate endocytosis into the cell and poorly expressed on healthy cells to deliver drugs to cancer cells [7].

### 2.3. Sources, Requirement and Deficiency Symptoms

Humans are unable to synthesize folic acid endogenously, therefore vitamin B9 must be fully supplied with food or supplemented with vitamin preparations [4,37]. The recommended daily intake of folic acid (Table 1) depends on age and, in women, on the physiological status (e.g., pregnancy, lactation) [37]. The requirement for folic acid may increase in certain pathologies and during the use of specific therapies, especially those that disrupt folic acid metabolism (e.g., cytostatics) [37,56,57].

Natural folic acid is commonly found (as various types of folates) in a wide range of foods (Table 2).

Natural dietary folates are less stable than synthetic folic acid (e.g., a component of drugs, supplements or fortified foods). Folic acid losses during harvesting, storage, distribution and culinary processing of food products can be significant. It is estimated that vitamin B9 loss during cooking of vegetables and fruit reaches as much as 50–80%. Dietary folates are also absorbed more slowly than synthetic folic acid, and their bioavailability depends largely on the type of food, meal composition and its general physicochemical characteristics (e.g., pH). It is estimated that 170 μg of folate is equivalent to 100 μg of synthetic folic acid. This means that the dietary equivalent of folate is its content in food multiplied by 1.7 [60]. Due to the widespread occurrence of folates in staple foods, despite their low stability and variable bioavailability in natural sources, it is believed that a diet containing at least three servings of fresh green vegetables per day should meet the daily requirement for vitamin B9 under physiological conditions. The exceptions are women who are pregnant, lactating or preparing for pregnancy [61].

Due to the widespread presence of folates in staple foods, folic acid deficiency is a rare phenomenon, although it is suspected that its scale may be underestimated. Most often, this condition results from insufficient intake (unbalanced diet), malabsorption, increased physiological demand or increased demand resulting from pharmacotherapy [38,62,63]. Folic acid deficiency induces inhibition of proliferation and interruption of the cell cycle. This leads to impaired cell proliferation and maturation and accelerates the aging process [48]. Clinically, folic acid deficiency leads to megaloblastic anemia [64,65,66], pancytopenia [67,68] and neuropsychiatric symptoms [69,70], including depressed mood [71,72] and cognitive impairment [73]. Folic acid deficiency may also contribute to complicated pregnancy, including neural tube defects in the fetus [74,75,76].

## 3. Hypersensitivity to Folic Acid and/or Folinic Acid

The first published clinical description of a hypersensitivity reaction to folic acid dates back to 1949 [77]. Since then, fewer than forty documented cases of allergic hypersensitivity to folic acid (Table 3) or folinic acid (Table 4) have been reported, with varying degrees of severity and symptom manifestations, including anaphylactic shock. Interestingly, most of these reactions occurred in women. Reactions were usually associated with folic or folinic acid supplementation for prophylactic purposes (in pregnant women and those planning a pregnancy), for the treatment of anemia or during cancer chemotherapy. No case of hypersensitivity to folates naturally occurring in food has been reported to date.

According to reported case reports, hypersensitivity to folic acid (Table 3) or folinic acid (Table 4) can have a variety of clinical presentations. Mild dyspeptic symptoms, a feeling of heat, hives of varying degrees of severity, localized (usually facial) or generalized edema, as well as severe anaphylactic reactions are possible.

An analysis of the reported folic acid hypersensitivity reactions (Table 3) highlights the fact that all events occurred after exposure to synthetic folic acid administered orally or intravenously in the form of various vitamin medications, vitamin supplements or foods fortified with synthetic vitamin B9. There are no reports of hypersensitivity to folates naturally occurring in food products. There have also been no reports of hypersensitivity following local exposure or allergic reactions associated with occupational exposure to folic acid (e.g., during industrial synthesis).

Folinic acid (Table 4) caused hypersensitivity reactions following intravenous administration. No such situation has been described after oral administration of this substance or after local exposure. Furthermore, all described cases (Table 4) involved folinic acid (leucovorin) administered during the treatment of cancer, usually colon cancer, and hypersensitivity reactions always required differentiation from a possible allergy to the chemotherapeutic agents used. It is also worth noting that, in addition to the symptoms typical of this type of clinical event (such as shortness of breath, swelling, redness or hives), symptoms associated with the nervous system (e.g., numbness in the extremities), pain limited to certain areas of the body (e.g., the back) or generalized pain were also observed.

The cause of the hypersensitivity reaction appears to be synthetic folic acid, which is a component of dietary supplements, medications or added to foods to enhance their nutritional value. Interestingly, however, folates naturally occurring in food products likely do not have allergenic properties.

## 4. Probable Mechanisms of Folic Acid or Folinic Acid Hypersensitivity

No definitive mechanism for hypersensitivity to folic acid or its derivatives (including folinic acid) has yet been described. The type and nature of reported clinical events (Table 3 and Table 4) suggest that more than one pathogenetic pathway may be involved in vitamin B9 allergy. Mixed mechanisms of folate hypersensitivity cannot be ruled out either [10,85,92,107].

### 4.1. Folic Acid

For example, Nucera et al. [92] described three different cases of folic acid hypersensitivity caused by oral supplementation with this vitamin (details in Table 3). The intensity and type of clinical symptoms, the course of the reaction and the results of diagnostic tests suggest different pathogenetic pathways for these events. It is likely that two of these cases were IgE-mediated, while the third was non-IgE-mediated and was classified by Nucera et al. [92] as a persistent drug eruption.

**Figure 4:**
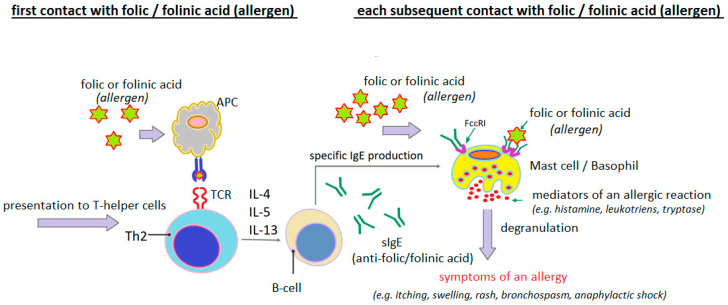
Probable mechanism of IgE-dependent hypersensitivity reaction to folic acid or folinic acid as a complete allergen (author’s own figure).

Analyzing the clinical cases of hypersensitivity reactions to folic acid presented in Table 3, it can be seen that in many of them, both the clinical course of the reaction and the results of the diagnostic tests performed indicate with a high degree of probability the IgE-dependent nature of these reactions (Figure 4).

**Figure 5:**
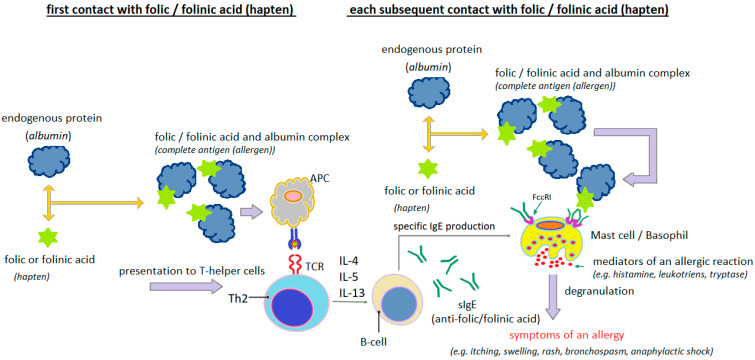
Probable mechanism of IgE-dependent hypersensitivity reaction to folic acid or folinic acid as a hapten (author’s own figure).

The presence of specific IgE antibodies to folic acid was demonstrated by Dykiewicz et al. [85] and Gaeta et al. [93]. Dykiewicz et al. [85] first detected IgE antibodies specific to the folic acid/human serum albumin (FA/HSA) conjugate in a woman who had experienced anaphylaxis twice after taking various multivitamin mixtures with folic acid. They then performed inhibition tests of the sIgE antibodies detected in the patient’s serum using HSA, FA and FA/HSA. Only the FA/HSA conjugate blocked the detected antibodies. Neither FA nor HSA used separately were effective. This suggests that FA acts as a hapten, acquiring the properties of a full antigen (allergen) only after binding to an endogenous protein (usually albumin) (Figure 5).

**Figure 6:**
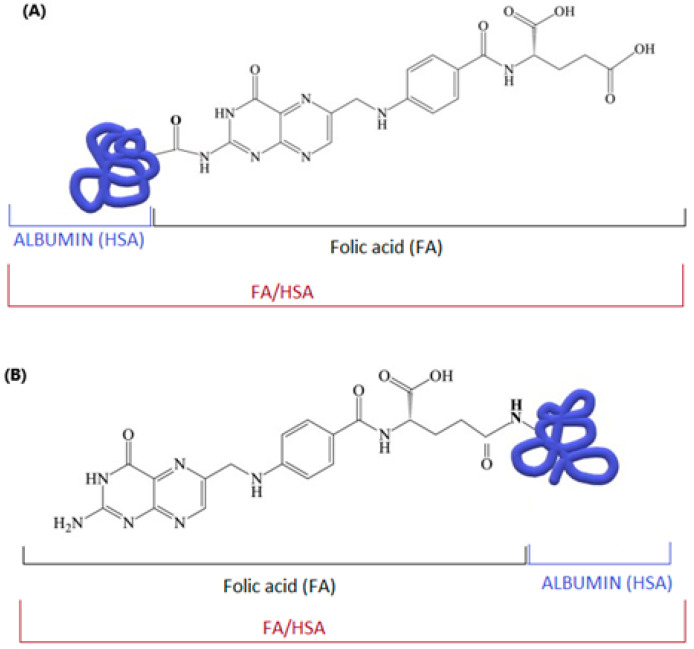
Possible structures of the “folic acid–albumin” conjugate: (**A**) via the amino group of folic acid and the carboxyl group of albumin; (**B**) via an amide bond between the carboxyl group of the folic acid residue and the amino group of albumin; author’s own figure based on [113,117,118].

Albumin is a transporter of folic acid in the blood. Various types of folic acid–albumin bonds are possible (Figure 6). The type and site of binding influence the stability of the entire complex and the conformation of albumin [110,111,112,113,114,115,116].

The hapten theory of folic acid is plausible, although there are also counterintuitive factors. According to Gaeta et al. [93], allergy to folic acid and/or its derivatives is likely associated with an IgE-dependent mechanism, in which the sensitizing factor is free (unbound to protein) folic acid or its derivatives. These researchers point to the lack of clear data confirming the phenomenon of endogenous haptenization of folic acid or its derivatives in human metabolism and the fact that free folic acid stimulates histamine release from basophils. Furthermore, as reported by Gaeta et al. [93], drugs with a similar molecular weight to folic acid can cause sensitization via an IgE-dependent mechanism without prior haptenization.

It is also noteworthy that, despite the widespread natural occurrence of folates in food, reactions to this group of compounds present in natural products have never been reported. Reactions that developed after consuming food were associated with the consumption of foods artificially enriched with synthetic folic acid [10,86,87,93]. It has also been noted that people sensitive to folic acid supplements can also consume high-folate foods (e.g., spinach) without any adverse reactions [91]. This situation is likely due to the fact that pteroylglutamic acid (folic acid) is almost always naturally present as polyglutamic acid, while pharmaceutical products, supplements and fortified foods use the monoglutamine form (pteroylmonoglutamate) [84,119].

Multi-stage folate metabolism occurs within enterocytes, where monoglutamate folic acid is reduced, methylated and released into the bloodstream as 5-methyltetrahydrofolate monoglutamate. This causes folates from natural sources to be absorbed very slowly (their bioavailability is low), while free folic acid (monoglutamate) does not penetrate into the plasma. Therefore, the concentration of folates entering the blood from natural sources may be too low to trigger such reactions [79]. Synthetic folic acid has a significantly higher bioavailability than natural folates and can be rapidly absorbed by enterocytes [120]. Monoglutamates delivered rapidly in high concentrations may exhaust the body’s capacity for methylation and reduction to 5-ethyltetrahydrofolate (due to saturation of the enzymatic systems involved in this process), resulting in the absorption of pteroylglutamic acid monoglutamates into the circulation [79]. Studies show that unmetabolized synthetic folic acid can be detected in the blood even at doses as low as 200 μg [10]. Assuming a hapten role for folic acid, these absorbed, unmetabolized monoglutamine molecules bind to endogenous proteins, becoming immunogenic and potentially causing immediate allergic reactions [84]. This mechanism seems particularly likely in the case of intensive supplementation with synthetic folic acid due to the saturation of gastrointestinal enzymes following excessive exposure to a high dose of folic acid [10].

### 4.2. Folinic Acid

The probable mechanisms of hypersensitivity to folinic acid (5-formyltetrahydrofolic acid, leucovorin) were analyzed by Apraxine et al. [107]. These researchers based their findings on the analysis of two clinical cases they reported and previously published reports (Table 4). They noted that hypersensitivity to folinic acid can likely occur via both IgE-dependent and -independent mechanisms. A mixed nature of hypersensitivity to leucovorin cannot be ruled out. Regardless of the pathogenetic mechanism of folinic acid hypersensitivity, the reaction rate may also be caused by the route of exposure. Folinic acid is usually administered intravenously, which means it is not subject to intestinal folic acid metabolism and is delivered rapidly at high concentrations, which may be an additional factor predisposing to the development of various adverse reactions, including hypersensitivity reactions [107]. It should be noted that investigating the mechanisms of hypersensitivity to folinic acid is significantly hampered by the fact that these reactions have so far occurred in individuals treated for cancer, primarily colorectal cancer (Table 4). Both the underlying disease and the treatment, which is very burdensome for the patient’s body, may influence the development of the immune response and predispose to the occurrence of various complications, including hypersensitivity reactions [121,122,123,124]. Furthermore, it should be noted that in the context of hypersensitivity reactions during cancer treatment, the therapeutic team’s attention is usually focused on the chemotherapeutic agents or biological drugs used [103], while other, less obvious drugs and adjuvants are often overlooked as unlikely factors that could cause allergy [107,125,126,127,128]. It also cannot be ruled out that hypersensitivity reactions develop independently to both the chemotherapy agent and folinic acid [109].

## 5. Hypersensitivity to Folic or Folinic Acid—Cross-Allergies to Folic Acid Analogues

**Figure 7:**
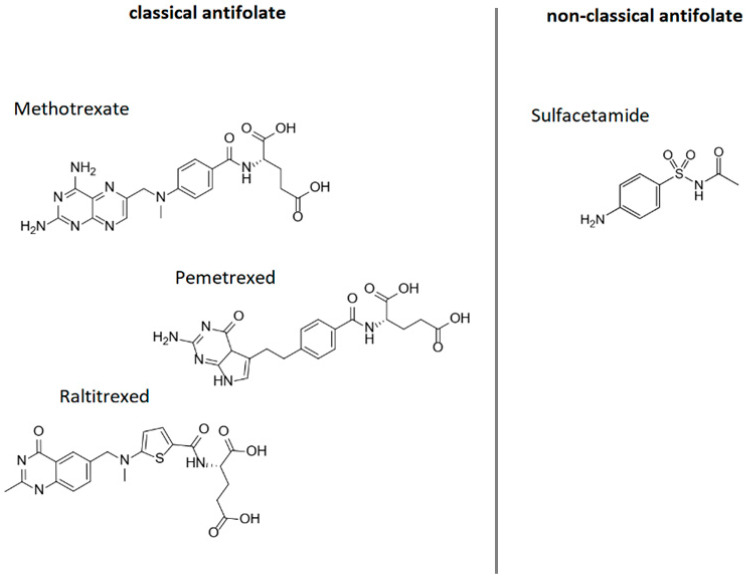
Folic acid analogues; author’s own figure based on [42,129,130,131,132].

Folic acid analogues (antifolates) are chemical compounds that have a structure similar to folic acid and different biological activity. Antifolates are classified as classic (e.g., methotrexate, pemetrexed and raltitrexed) and non-classic (e.g., sulfonamides) (Figure 7).

These substances are also called antimetabolites. Antimetabolites inhibit enzymes involved in the synthesis of nitrogenous bases (e.g., dihydrofolate reductase (DHFR), a key enzyme in folic acid metabolism) by competing with folic acid. Blocking DHFR stops the conversion of folic acid to its active form, tetrahydrofolic acid, which ultimately results in the inhibition of DNA and RNA synthesis. Interrupting DNA replication prevents further cell division and results in, among other things, inhibiting the proliferation of cancerous tissue. In medicine, folic acid analogues are mainly used as anticancer and immunosuppressive drugs [42,129].

Available literature data indicate that antifolates, especially the classic ones, should be considered as a probable factor causing hypersensitivity reactions, in the mechanism of cross-reactivity, in people allergic to folic acid or its derivatives, which results from the similarity in the structure of a significant fragment of the molecule [10,85,90,93].

Nishitani et al. [90] described the case of a 42-year-old woman who developed generalized urticaria and shortness of breath 2 h after taking a folic acid supplement. The patient had never experienced such symptoms before, although she had taken folic acid preparations approximately 2 years earlier. The patient underwent skin prick testing with the suspected supplement and its components: folic acid (5 mg/mL), riboflavin, biotin, niacin, thiamine, pyridoxine hydrochloride, calcium pantothenate and yeast. Skin prick testing was also performed with folic acid analogues: methotrexate (2 mg/mL) and folinic acid (1 mg/mL). Positive reactions were found to the vitamin supplement, folic acid and methotrexate, but not to folinic acid. These researchers also performed a histamine release test (HRT) with folic acid, folinic acid and methotrexate, obtaining positive results for folic acid and methotrexate, but not for folinic acid. It therefore appears that cross-hypersensitivity reactions between folic acid and its analogues are possible, although not necessarily with all derivatives. Similar conclusions are provided by the results of inhibition tests performed by Gaeta et al. [93], who observed that IgE antibodies specific for folic acid are blocked by both folic acid (strong inhibition) and methotrexate (moderate inhibition). Studies by other authors [10,85,93] also seem to confirm that the phenomenon of allergic cross-reactivity to methotrexate in individuals initially allergic to folic acid is possible and may have broad clinical implications. Gaeta et al. [93] suggest that patients with a history of folic acid or folate allergy should be considered at increased risk of hypersensitivity to methotrexate and other classic antifolates (e.g., pemetrexed), while patients with immediate-type reactions to methotrexate and pemetrexed may be at increased risk of reaction to synthetic folic acid found in dietary supplements and fortified foods.

There is no data on cross-sensitivity to non-classic antifolates (e.g., sulfonamides). This group of drugs can cause hypersensitivity reactions [133,134]; however, allergy to folic acid has not yet been considered as a potential cause of allergic response after exposure to sulfonamides.

## 6. Hypersensitivity to Folic and/or Folinic Acid—Current Diagnostic Options

Diagnosing hypersensitivity to folic acid or folinic acid is fraught with difficulties, stemming in part from the fact that these reactions are rare. This means that folates are not considered as possible causative factors at the beginning of the diagnostic process.

A significant methodological problem in the diagnosis of folate hypersensitivity is the lack of standardized tests for both in vitro and in vivo testing. Although the presence of sIgE antibodies to folic acid has been described, these tests were performed using proprietary immuno(dot)blot and ELISA (enzyme-linked immunosorbent assay) assays [85,93]. Commercial, validated kits for detecting folic acid-specific IgE are not available.

It is noticeable that the diagnostic process in clinical cases reported by various authors (Table 3 and Table 4) typically involved various types of skin tests (prick and intradermal) with available pharmaceutical preparations of folic acid and/or its derivatives. This strategy appears to be the most accessible diagnostic test when folate hypersensitivity is suspected based on a carefully collected clinical history.

The basophil activation test (BAT) appears to be a good solution for diagnosing allergy to folic acid or its derivatives. This procedure was successfully used by Gouveia et al. [96]. This technique appears to be very promising; however, the lack of a folic acid solution validated for the BAT test and the lack of reference values significantly limits its use in routine diagnosis of hypersensitivity to folic acid or its derivatives.

It appears that the introduction of standardized diagnostic procedures and the development of validated diagnostic tools (e.g., folic and folinic acid solutions) for in vivo diagnostics (such as skin prick tests, epidermal patch tests, provocation tests) and in vitro diagnostic kits (e.g., determination of folate-specific IgE and BAT) are necessary to significantly improve and enhance the diagnostics of sensitivity to folic acid or folinic acid. This, in turn, may increase the diagnostic effectiveness of folate hypersensitivity, favorably impacting appropriate therapeutic management in individuals who require folate supplementation, and support understanding of the pathogenic mechanisms of vitamin B9 hypersensitivity.

## 7. Summary

According to available data, hypersensitivity to folic acid and/or folinic acid is a relatively rare phenomenon. Clinically, these reactions have a very diverse range of manifestations. They range from self-limiting, mild symptoms (usually generalized or localized sensations of heat, hives or itching) to severe anaphylactic reactions, which occur sporadically and are more likely to occur after intravenous administration of vitamin B7. The spectrum of symptoms of folinic acid hypersensitivity has also included neurological symptoms (e.g., impaired sensation in the legs) or pain (e.g., back pain). It is worth noting that reactions can occur after both oral and intravenous administration. Intravenous administration is more likely to involve folinic acid, but this may be due to the specific therapy in which this derivative is used (often in combination with chemotherapy in cancer treatment). It is also worth noting that folates, naturally found in food, are unlikely to cause hypersensitivity. Reactions that have developed as a result of consuming various food products have always been associated with the consumption of foods fortified with synthetic folic acid or multivitamin drinks.

The mechanism of hypersensitivity reactions to folic or folinic acid is not entirely clear. Both IgE-dependent immediate reactions and other non-IgE-mediated pathomechanisms have been implicated. It is also unclear whether folic acid is a standalone allergen or whether it functions as a hapten, acquiring the characteristics of a full-fledged antigen only after binding to endogenous proteins. It appears that intensive intake of synthetic folic or folinic acid during supplementation may be a significant predisposing factor to hypersensitivity to these substances.

Hypersensitivity to folic or folinic acid should be considered when diagnosing unclear allergic reactions with a difficult-to-determine causative agent, associated with the use of vitamin preparations, vitamin-fortified foods, combination therapies involving folates and in situations suggesting the possibility of a hypersensitivity reaction due to cross-reactivity (e.g., during methotrexate therapy).

Diagnosing folate hypersensitivity is difficult primarily due to the lack of standardized diagnostic tests for detecting folate-specific IgE antibodies and the lack of commercially available pure folic acid solutions for skin testing or allergen challenge in BAT. BAT appears to be a very promising tool in diagnosing folate hypersensitivity, but the stimulation solutions, the procedure and the clinical interpretation of results require standardization.

## Figures and Tables

**Table 1:** Recommended daily intake of folic acid according to the recommendations of the Food and Nutrition Board (FNB) at the National Academies of Sciences, Engineering, and Medicine [58].

Age	Male	Female
0–6 months	65 mcg DFE	65 mcg DFE
7–12 months	80 mcg DFE	80 mcg DFE
1–3 years	150 mcg DFE	150 mcg DFE
4–8 years	200 mcg DFE	200 mcg DFE
9–13 years	300 mcg DFE	300 mcg DFE
14–18 years	400 mcg DFE	400 mcg DFE
>19 years	400 mcg DFE	400 mcg DFE
Female-Pregnancy		400 mcg DFE
Female-Lactation		500 mcg DFE

Legend: DFE-dietary folate equivalents; 1 mcg DFE = 1 mcg food folate; 1 mcg DFE = 0.6 mcg folic acid from fortified foods or dietary supplements consumed with foods; 1 mcg DFE = 0.5 mcg folic acid from dietary supplements taken on an empty stomach.

**Table 2:** Content of natural folates in selected food products [59].

Product	Fresh [μg/100 g]	Cooked [μg/100 g]	Cooking Loss (%)
Wheat bran	258		
Whole wheat bread	39		
Broad beans	130	37	72
Peas	87	34	61
Brussels sprouts	88–170	36	69
Cauliflower	120	51	58
Broccoli	90	64	29
Asparagus	70–175		
Spinach	155	29–90	42
Cabbage	16–45	11	53
Lettuce	33		
Paprika	37		
Bananas	28–36		
Oranges	24		
Apples	6		
Liver	219–364	145–240	34
Beef, poultry, pork	5–18		
Eggs	70	30	57
Cheese	16		
Cow’s milk (fresh)	2–12		

**Table 3:** Hypersensitivity to folic acid in clinical cases (based on available data, reference numbers are in the table).

Author(s)/Year of Publication	Patient: Sex/Age	Clinical Symptoms	Product Type/Route of Administration	Diagnostics
Mitchell, D.C. et al./1949 [77]	F/35 y.	(1 episode) itchy rash after two weeks of therapy disappeared after discontinuing the drug	FA/p.o.	IDT (FA): positive
		(2 episode)—anaphylactoid reaction (dyspnea, tachycardia, anxiety, flush)—reaction disappeared spontaneously	FA/i.v.	
Raush, F./1956 [78]	ND/ND	anaphylactic shock	FA/p.o.	ND
Chanarin, I. et al./1957 [79]	M/35 y.	General malaise, aching pain in lower thoracic region, respiratory difficulty with restricted inspiration, itching of palms of the hands and soles of the feet, generalize pruritus with eruthrematous rush—reaction disappeared spontaneously within three hours	FA/p.o.	IDT (FA): positive IDT (FAA): positive IDT (FnA): negative IDT: (FAC): negative
Levander-Lindgren, M./1957 [80]	F/ND	Anaphylactic shock	FA/ND	ND
Woodlif, H.U. and Davis, R.E./1966 [81]	F/36 y.	General feeling of unwellness, hives (two episodes)	FA/p.o.	IDT (FA): positive IDT (FnA): positive IDT (AM): positive IDT (MX): negative
	F/24 y.	General feeling of unwellness, urticaria	FA/i.v.	
Mathur, B.P./1966 [82]	M/9 m.	Generalized urticaria (two episodes)	FA/p.o.	ND
Sesin, G.P. and Kirschenbaum, H. /1979 [83]	M/36 y.	Generalized pruritus—symptoms resolved after treatment was stopped and returned when treatment was restarted three months later	FA/p.o.	IDT (FA): positive
Sparling, R. and Abela, M./1985 [84]	M/62 y.	Severe hypersensitivity reaction: bronchospasm, generalized itchy rash (two episodes)	FA/p.o.	IDT (FA): positive
Dykewicz, M.S. et al./2000 [85]	F/32 y.	Urticaria, facial angioedema, nausea and repeated vomiting	MV/FA/p.o.	sIgE (FA/HSA): positive SPT (FA): positive SPT (FoA): positive SPT (M-THF): positive SPT (THF): positive SPT (MX): negative OBCT (FoA): positive
Sanders, G.M. and Fritz, S.B./2004 [86]	F/61 y.	Anapxylaxis (urticaria, recurrent angioedema)	MV/FA/p.o. FF-FA/p.o.	SPT (FA): positive SPT (Bvit): negative SPT (Cvit): negative SPT (ADEK): negative OBCT (FA): positive
Smith, J. et al./2007 [87]	F/ND	(1 episode) itchy throat, nausea, generalized rash, diarrhea and dizziness	FA/p.o.	IDT (FA): positive OBCT (FA): positive
		(2 episode) itchy throat, generalized itching and nausea	W-FA/p.o.	
		(3 episode) generalized rash, vomiting and dizziness	Sup-FA/p.o.	
Pfab, F. et al./2007 [88]	F/44 y.	Anaphylactic reaction with tachycardia, generalized skin eruption and dyspnea (4 episodes)	MV/FA/p.o	SPT (MV/FA): positive SPT (FA): positive SPT (B12): negative SPT (B6): negative OBCT (MV/FA): positve
Valdivieso, R. et al./2009 [89]	F/72 y.	Urticaria (daily for several hours) and occasional facial angioedema (symptoms for 8 months)	FA/p.o.	SPT (FA): positive
Nishitani, N. et al./2009 [90]	F/42 y.	Generalized hives and shortness of breath	FA/p.o.	IgE(t): normal value SPT (Sup): positive SPT (FA): positive SPT (Bvit): negative SPT (MX): positive SPT (FnA): negative HRT (FA): positive HRT (MX): positive
Roy, S. and Roy, M./2012 [91]	F/29 y.	Generalized itchy maculopapular rash (9 weeks pregnant)	MV/FA/p.o.	Not conducted
Schrijvers, R. et al./2015 [10]	F/53 y.	Anaphylactic shock (facial angioedema, nausea, diarrhea, dyspnea with desaturation and hypotension	FA/p.o.	SPT (sup): positive SPT (FA): positive SPT (MV/FA): positive SPT (B1-B6): negative OBCT (B1-B6): negative SPT (FoA): negative SPT (MX): negative
		Pruritus, flush, diarrhea and need to lie down to recover	FF-FA	
Nucera, E. et al./2018 [92]	F/47 y.	Generalized urticaria (2 episodes day after day)	FA/p.o.	SPT (FA): positive IDT (FA): positive
	M/66 y.	Acute urticaria and loss of consciousness	FA/p.o.	SPT (FA): positive OBCT (FA): positive
	F/40 y.	Round erythematous–violaceous well-defined macula on the left iliac spine associated with itching and burning	FA/p.o.	SPT (FA): negative IDT (FA): negative OBCT (FA): positive
Gaeta, F. et al./2020 [93]	F/30 y.	(1 episode) anaphylactic reaction with urticaria, dyspnea, tachycardia, hypotension and lipothymia	FA/p.o.	SPT (FA): positive IDT (M-THF): positive IDT (FoA): positive IDT (MX): positive sIgE (FA): positive IT (FA): strong positive IT (MX): positive IT (M-THF): week positive
		(2 episode) extensive erythematous rush	FF-FA/p.o.	
	F/34 y.	(1 episode) angioedema, vomiting, dyspnea, lipothymia and spontaneous resolution after 40 min.	FA/p.o.	IDT (FA): positive
		(2 episode) more severe reaction with angioedema, dyspnea, hypotension, tachycardia and lipothymia	FA/p.o.	
Schnebert, B./2023 [94]	F/49 y.	Anaphylactic reaction (Grade 2 according to the Ring and Messmer’s classification) manifesting with urticaria, angioedema, dyspnoea and dysphonia (intramuscular adrenaline, oral corticosteroids and antihistamines	FA/p.o.	IDT (FA): positive SPT (FA): positive Tryptase: 40.8 mU/L (normal <6.48 mU/L)
Nonhoff, J. et al./2023 [95]	F/60 y.	(1 episode) tingling in the legs, generalized urticaria, itching, increasing shortness of breath	FA/p.o.	SPT (FA): positive SPT (MVJ/FA): positive SPT (L-MF): positive SPT (CL-MF): positive SPT (M-THF): positive
		(2 episode) generalized urticaria, itching, increasing shortness of breath	MVJ/FA	
Gouveia, J. et al. /2024 [96]	F/38 y.	Palmoplantar pruritus, generalized urticaria, angioedema and conjunctival hyperemia	FA/p.o.	SPT (FA): positive BAT (FA): positive

Legend: F—female; M—male; y.—year; m.—month; ND—no data; p.o.—orally (latin “per os”); i.v.—intravenously (latin “ iniectio intravenosa”); IDT—Intradermal Tests; SPT—Skin Prick Tests; OBCT—oral blind challenge test; tIgE—total Immunoglobulin E; sIgE—specific Immunoglobulin E; IT—Inhibition test; HRT— histamine release test; BAT—basophil activation test; FA—Folic Acid; FAA—Folic Acid Analogue FoA—Folinic acid; FAC—Folic Acid Conjugat; FF-FA—food fortified with folic acid; Sup-FA—folic acid suplement; W-FA—wather fortified with folic acid; Bvit—witaminy B (różne); Cvit—witamina C; ADEK—witaminy A, D, E, K; MX—metotrexat; AM—aminopteryna; THF—tetrahydrofolate; M-THF—5-methyl-tetrahydrofolate; MV/FA—multiwitamin wih folic acid; MVJ/FA—multiwitamin juice wih folic acid; L-MF—L-Methylfolate; CL-MF—Calcium L-Methylfolate; B12—vitamin B12; B6—vitamin B6; B1-B6—mieszanina witamin od B1 do B6; FA/HSA—folic acid/human serum albumin; Sup—supplement “as is”.

**Table 4:** Hypersensitivity to folinic acid in clinical cases—leucovorin was administered intravenously (i.v.) as a supplement to cancer treatment with chemotherapeutic agents (based on available data, reference numbers are in the table).

Author(s)/Year of Publication	Patient: Sex/Age	Clinical Symptoms	Diagnostics
Benchalal, M. et al. /2002 [97]	M/80 y.	(1 episode) urticaria	Not conducted
		(2 episode) urticaria, profound hypotension	
Vermeulen, C. et al./2003 [98]	M/57 y.	Urticaria, difficulty breathing	SPT (FoA): positive IDT (FoA): positive SPT (FA): negative
	M/59 y.	(1 episode) urticaria	SPT (FoA): negative IDT (FoA): positive
		(2 episode) urticaria	
Prabu, R. and Bakhshi, S./2009 [99]	M/16 y.	(1 episode) chills and rigors, followed by erythematous rash over face, neck and upper limbs along with a temperature spike of 38.8 °C (after second dose of leucovorin)	tIgE: 711 IU/mL (normal: 0.0–158 IU/mL)
		(2 episode) generalized erythematous rash that blanched on pressure, vomiting, dizziness and hypotension (after third dose of leucovorin)	
Katirtzoglou, N.A./2011 [100]	F/67 y.	(1 episode) feeling of heat, facial redness, cough, shortness of breath, hypertension and vomiting	Not conducted
		(2 episode) redness, burning sensation all over the body and severe diarrhea (without shortness of breath or drop in blood pressure)	
		(3 episode) facial flushing, feeling of heaviness in the chest, shortness of breath, vomiting, hypotension (90/60 mm Hg)	
Damaske, A. et al./2012 [101]	M/53 y.	(1 episode) flushing, uricaria, pruritus	Not conducted
		(2 episode) urticaria	
		(3 episode) headache, generalized pain, facial flushing	
		(4 episode) generalized discomfort, headache, back pain, pruritus, dyspnea	
		(5 episode) facial flushing, headache, severe lower back pain, hypertension (227/114)	
Ureña-Tavera, A. et al./2015 [102]	M/65 y.	Facial erythema and general urticaria (30 min after)	SPT (FoA):negative DPT (FoA): positive
	F/66 y.	(1 episode) limited general itching	SPT (FoA):negative DPT (FoA): positive
		(2 episode) genital and scalp itching, rhinoconjunctivitis and general malaise (50 min after)	
	F/52 y.	(1 episode) mild chills	SPT (FoA):negative DPT (FoA): positive
		(2 episode) intense chills (70 min after)	
	F/73 y.	Facial erythema, general urticaria and eyelid angioedema (20 min after)	SPT (FoA):negative DPT (FoA): positive
	F/80 y.	(1 episode) limited chest pain	SPT (FoA):negative DPT (FoA): positive
		(2 episode) dyspnea, chest pain, oxygen desaturation (85%) and facial erythema (10 min after)	
Florit-Sureda, M. et al./2016 [103]	M/56 y.	(1 episode) generalized redness, itching, erythema and oedema of the face and chest and abdominal pain	Not conducted
		(2 episode) facial erythema, dyspnea and pruritus	
		(3 episode) facial erythema, dyspnea and pruritus	
Mathew, A.A. et al./2017 [104]	F/2 y.	(1 episode) itching over forehead, but no rashes or features (42 h after the first dose)	Not conducted
		(2 episode) erythematous rashes over faces neck, back of trunk, both upper limbs with ptosis secondary lid edema (after second dose)	
		(3 episode) erythematous rashes over faces neck, back of trunk, both upper limbs with ptosis secondary lid edema (after third dose)	
Nesbitt, P. et al./2019 [105]	M/65 y.	(1 episode) acute back and knee pain, diaphoresis, anxiety and nausea	Not conducted
		(2 episode) acute back and knee pain, diaphoresis, anxiety and nausea—intensification of symptoms	
Gudimetla, V. et al./2019 [106]	F/62 y.	Numbness in legs and chest (three episodes)	Not conducted
Apraxine, M. et al./2022 [107]	M/72 y.	(1 episode) lower back muscule pain	Tryptase: 16 μg/L (normal: <14 μg/L) SPT (FoA):negative IDT (FoA): positive
		(2 episode) chills an facial oedema	
		(3 episode) facial and chest erythema and chills	
		(4 episode) diffuse erythema with labial oedema	
	F/45 y.	(1 episode) lower back muscule pain and chills	Tryptase: 8.1 μg/L (normal: <14 μg/L) SPT (FoA):negative IDT (FoA): negative
		(2 episode) lower back muscule pain and chills	
		(3 episode) chills, cyanosis, tachycardia	
Kim, M. et al./2023 [108]	F/60 y.	(1 episode) facial flushing and pruritus on the trunk, hypotension (90/50 mmHg, pulse 101 beats per minute (bpm), peripheral capillary oxygen saturation (SpO2) 89%) (after 30 min)	Tryptase (4 h after the incident): 11.4 μg/L (normal: <11 μg/L)
		(2 episode) facial flushing and dyspnea, hypotension (96/60 mmHg), pulse 103 beats bpm and SpO2 100% (after 20 min)	Not conducted
		(3 episode) dyspnea, urticaria (after 5 min)	Not conducted
		(4 episode) facial redness, hypotension (100/68 mmHg), pulse 86 bpm, SpO2 97% (after 20 min)	Not conducted
		(5 episode) dyspnea, redness of the trunk, hypotension (100/60 mmHg), SpO2 of 95% (after 30 min)	Not conducted
		(6 episode) facial redness, dyspnea, hypotension (96/64 mmHg), pulse 74 bpm, SpO2 88% (after 15 min)	Not conducted
Muzaffar, A. and Alvarez Arango, S./2024 [109]	F/51 y.	Severe abdominal pain and erythematous spots on the face and trunk (symptoms recurring with each leucovorin infusion)	IDT (FoA): positive

Legend: F—female; M—male; y.—year; i.v.—intravenously (latin “iniectio intravenosa”); IDT—Intradermal Tests; SPT—Skin Prick Tests; DPT—drug provocation test; tIgE—total Immunoglobulin E; FoA—Folinic acid.

## Data Availability

Not applicable.
